# Encouraging help-seeking and engagement in a mental health app: What young people want

**DOI:** 10.3389/fdgth.2022.1045765

**Published:** 2022-12-21

**Authors:** Sandra Garrido, Eliza Oliver, Anthony Chmiel, Barbara Doran, Katherine Boydell

**Affiliations:** ^1^ The MARCS Institute for Brain, Behaviour & Development, Western Sydney University, Sydney, NSW, Australia; ^2^ Faculty of Transdisciplinary Innovation, University of Technology, Sydney, NSW, Australia; ^3^ Black Dog Institute, Sydney, NSW, Australia; ^4^ Faculty of Medicine, University of New South Wales, Sydney, NSW, Australia

**Keywords:** mental health app, MoodyTunes, young people, adolescents, music listening, app engagement, focus group, mood disorder

## Abstract

Although many young people evince a preference for digital mental health support over face to face, engagement with smartphone apps for mental health remains relatively low, particularly in young people not accessing professional support services. While some of this can be attributed to stigma or embarrassment, there is also a need for developers and researchers to investigate features which promote long-term usage. *MoodyTune*s is a music-based smartphone app that has been co-designed with young people to help them develop effective self-management strategies for mood through music listening. Four focus groups with young people (*N* = 24, aged 13–25) were conducted to workshop how *MoodyTunes* could promote help-seeking behaviours and long-term engagement with the app. Online discussions following a broad topic guide were held in groups of 4–7 in which participants explored topics including the use of social features, promoting help-seeking behaviour, gamification and mood tracking. Participants also viewed visual materials and offered ideas about visual design both verbally and on paper. A general inductive approach was taken to analysis of qualitative data. Three primary themes were identified in the data: privacy; user empowerment; and engagement vs. achievement. Participants voiced a strong desire to remain anonymous and to feel empowered to make their own decisions about accessing professional help. Sensitive use of language was noted as critical, with some participants noting that the use of more directive language could have a negative impact on their mental health and that motivational features should focus on engagement with the primary aim of the app rather than rewarding achievement. Findings highlight the benefits of a co-design approach and provide key insights into how mental health apps may be able to improve long-term user engagement with young people.

## Introduction

Mood disorders have been increasing around the world since 2010 (1, 2), particularly in young people (3). In the United States, major depressive episodes and suicidal ideation are estimated to now impact more than 13% of those aged 12 to 25, compared to approximately 8% in the decade prior (3). Despite this growing concern, only around 20% of young people in need receive professional support (4, 5).

There are multiple reasons for these low rates of professional help-seeking, with stigma and discrimination, embarrassment, and negative perceptions of mental health services being paramount (6). However, given that suicide is one of the leading causes of death in young people around the world (7, 8) there is a dire need to promote help-seeking behaviour in vulnerable young people. A key aspect of this is reducing the stigma surrounding mental health. While cultural changes in this area are slowly taking place, it is crucial that current programs and interventions encourage young people to reach out for assistance in a way that accommodates their concerns about stigma and embarrassment. Digital mental health interventions such as smartphone apps offer one solution, and many young people report preferring such options over face to face support (9, 10).

However, long-term engagement with mental health apps tends to be low (11), especially for males (12). One systematic review of smartphone apps for anxiety found that even apps with high user ratings tend to have declining engagement over time (13). It may be that the need for app engagement declines if users feel they have successfully gained skills in self-management of mood (14). Nevertheless, some users may benefit from sustained use of mental health apps in order to reinforce helpful mood management strategies.

The Persuasive System Design framework (15) proposes four mechanisms by which user engagement can be promoted in app design including: (1) facilitating the primary aim of the app; (2) promoting user interactions with the app; (3) leveraging social relationships; and (4) demonstrating credibility. However, some studies report mixed opinions on features often used to increase engagement *via* those mechanisms in health apps, such as social and competitive features, push notifications, or data tracking graphs (14). Furthermore, users can have an additional layer of concern about privacy in relation to mental health apps that may not exist to the same degree with other apps (16, 17). Prior work has demonstrated mixed attitudes towards the incorporation of social features in mental health apps by young people for this reason (18). However, other work has suggested that engagement tends to be higher in mental health apps that offer peer support (19) or other forms of social connection (20). These mixed findings indicate that these are issues to be negotiated with great care and extensive input from end-users in relation to mental health apps for young people.

*MoodyTunes* is a smartphone app which draws on principles of cognitive behavioural therapy and experiential learning in the context of music listening to help young people become more aware of how behaviours can influence their moods and wellbeing, as well as to help them develop effective strategies for self-management of mood. *MoodyTunes* was designed to be a more subtle approach to increasing mental health literacy and promoting effective mood management than many other apps, which often primarily rely on learning modules (21). The app works in the background as young people are listening to music on Spotify, prompting them to record the effect particular pieces of music are having on their mood, then creating automatic playlists of “feel better” music, in an effort to challenge habitual listening habits and increase awareness of what helps and what doesn't. The app also prompts young people to explore the thoughts and feelings being triggered by music while learning strategies such as cognitive reframing and challenging automatic negative thoughts.

*MoodyTunes* was developed using principles of co-design, with young people closely involved in the process from start to finish. In a first wave of focus groups, young people identified what they liked and disliked about currently available mental health apps (18) and generated ideas about how research about music and wellbeing could be transformed into a smartphone app. Then a small group of young people with app development experience produced a mock-up of the proposed app, which was then used as the basis of concept testing with 87 young people (22). Findings revealed that the app concept and its proposed features were enthusiastically received by users, but the need for an attractive visual design and clean user interface was identified. After the development of an initial prototype, a think-aloud study that included automated facial analysis data was conducted with 20 young people (23). Study findings revealed that the app was positively received, with a favourable rating being given to the ability to link to mental health resources, track mood over time and to share music that has helped. However, a need to improve the aesthetic appeal and ease of use of the app was further reiterated. In addition, the research team and government stakeholders involved in development of *MoodyTunes* wanted to include greater functionality for promoting help-seeking behaviours in a second iteration of the app.

The current study reports on a series of co-design workshops with young people to develop a second iteration of *MoodyTunes*. The study aimed to investigate young people's perspectives on how help-seeking behaviours could be promoted in a mental health app designed to appeal to those who may be averse to seeking professional assistance, as well as how to promote long-term engagement with the experiential learning processes included in the app.

## Materials & methods

### Study design

Workshops in the style of focus groups were conducted with young people to allow examination of shared experiences and synergistic idea generation whilst allowing individual perspectives and ideas to be heard (24). A general inductive approach was taken (25) in which findings were data-driven rather than based on *a priori* hypotheses, but were not designed to generate theory as in other inductive approaches such as grounded theory (26). A realist paradigm informed the research, taking a middle ground between constructivism and positivism, in that we aimed to give voice to individual experiences while attempting to derive a broad understanding of patterns of perspectives (27).

### Participants

Young people (*N* = 25, 15 females and 10 males) were recruited from a university in NSW, Australia and the broader public. To be eligible participants had to be between 13 and 25 years of age. Our previous research had involved young people within this age range and indicated few differences in response between younger participants and older ones (23). Potential participants were excluded if they demonstrated current suicidal ideation and had scores on the depression subscale of the Depression Anxiety Stress Scale (DASS-21 28); greater than 14, suggesting the presence of extremely severe depression. The reason for this exclusion is that *MoodyTunes* is not designed as a crisis management tool, and it would reduce risk to potential participants who required more critical care at the time of the study. One person was excluded on this basis and referred to professional support leaving a total sample of 24, (*n* = 14 participants <18 years of age, *n* = 10 participants 18+ years; see Table 1). A number of participants had been diagnosed with depression or anxiety or had self-identified as experiencing these conditions, but this was not a criterion for inclusion in the study and specific data about this was not linked to individual responses. Pre-screening data indicated that 6 participants (25%) were experiencing mild depression and 5 participants (20.8%) were experiencing severe depression at the time of the study.

**Table 1:** Summary of demographics for the four participant groups, *N* = 24.

Group number	Age group	Gender
G1, *n* = 7	<18	4 female; 3 male
G2, *n* = 4	18+	4 female
G3, *n* = 7	18+	5 Female; 2 male
G4, *n* = 6	<18	1 female; 5 male

Participants were allocated to one of 4 groups on the basis of time preference, although an attempt was made to balance genders across groups to the degree possible. We also attempted to keep people of similar ages in the same groups where practical, to ensure that younger participants felt as comfortable as possible expressing their opinions.

### Procedures

Ethics approval was obtained from the University ethics committee prior to commencement of the study (Approval number H14544). University students (*n* = 7, Group 3) were recruited *via* an internal study recruitment system and were offered course credit for participation. Other participants were recruited *via* notices on social media and emails sent to mailing lists of people who had previously expressed interest in being involved in future research. Participants who were not offered course credits received a $30 AUD digital voucher for participation. Some recruitment also occurred by word of mouth, with some participants inviting friends to join the study. Potential participants contacted the researchers and were screened for eligibility by email. Eligible participants were then emailed an information sheet and provided written consent or dual consent with parents for those under 16 years of age.

Participants attended an online workshop of approximately one hour conducted *via* Zoom due to health restrictions in place at the time in response to the COVID-19 pandemic. While some participants were known to each other, none were known to the group facilitator prior to the commencement of the study. Participants were advised that they were free to withdraw from the study at any stage, and that they were also free to have their camera either on or off according to their preference. Discussions then proceeded using a general topic guide designed to prompt conversations about social features, promoting health-seeking behaviour, gamification and mood tracking displays. Exploration of those topics by participants was allowed to proceed naturally once prompted, and with particular care taken to draw out divergent perspectives. Participants also viewed visual materials (described below) and were encouraged to offer their own design ideas throughout, whether drawn or described verbally. The data were also supplemented with notes taken by the group facilitator during the workshops based on participant responses, as well as any notes and drawings provided by participants.

### Materials

PowerPoint slides were used within Zoom using the “Share screen” function to display screenshots of the *MoodyTunes* prototype. An overview of the app and its features were firstly described to participants. Screenshots of various currently available mental health apps and design ideas for *MoodyTunes* were then used to elicit feedback and novel ideas about design issues (see **Supplementary Materials**). These screenshots included visual examples of features for accessing emergency health resources, graphs for displaying and tracking mood over time, and examples of gamification or reward systems.

### Data analysis

Workshop discussions were transcribed verbatim using inbuilt transcription features in Zoom which were checked against recordings by a member of the research team. Memos were also taken at this stage about group dynamics evident in the recordings in order to provide insight into ideas provoking broad consensus or conflicting viewpoints (29). Thematic analysis (30) then proceeded, starting with an initial wave of coding conducted independently by two authors. Incidental conversations that did not relate to the research aims of determining how to improve help-seeking behaviour and engagement was excluded from data analysis. Once consensus was reached, a second wave of analysis using axial coding as described by Charmaz (31) was performed. In this process codes from the first wave of analysis begin to be connected and organized in order to develop themes and subthemes. This was a collaborative process conducted primarily between two authors with consultation with the other authors and constant reference to the data. Three primary themes with sub-themes were derived from the data in an inductive process (Table 2) and are discussed in the following section.

**Table 2:** Themes, sub-themes and supporting quotes.

Theme	Sub-theme	Supporting Quote
Privacy	Data usage	“Clarifying what happens with data and not just in fine print, but actually explaining to a user with mental health might be a very good idea” (P5, G3, Female, 18+)
	Anonymity	“Maybe the first … two, three times, let them log in with like a guest account … to build a confidentiality … that nothing is being tracked or transferred from one place to another” (P5, G3, Female, 18+)
User empowerment	Retaining choice	“Having a pop-up saying, “we want you to talk to someone”, would make it feel much more aggressive than ‘here’s a little icon if you want more help’” (P1, G2, Female, 18+)
	Gentle and sensitive language	“If the wording of the notification makes it seem a bit more friendly, a bit more passive, so it seems like they still have the choice” (P3, G3, Male, 18+)
Engagement vs. achievement		“I think if I were to use the app I wouldn’t really care about rewards or anything like that. I would just use the app to up my moods” (P4, G1, Female, <18)
		Female

## Results

Overall, participants agreed that the *MoodyTunes* app concept was appealing and likely to be something that could help some young people become more conscious of the effect of music on their mood. As one participant stated:

I think this app could be really cool for people who are a bit out of touch with themselves and their mood. Because you go through life, do your job and see your friends, but you're go, go, go and you don't really have the time to reflect. You may be happy, whatever this means, but I think this app could be really cool if it not only helped during those depressive times but is a bit of a record of all your moods. (P3, G2, Female, 18+).

Similarly, a second participant noted that the app “could definitely help put [mental health] in context and make you realise, you know, this isn’t just a bad mood, this is something that I need to consider getting help for” (P4, G4, Female, <18).

In relation to app features to encourage help-seeking behaviour and user engagement, three primary themes were identified: (i) privacy, (ii) user empowerment, (iii) engagement vs. achievement.

### Privacy

Privacy was an issue that was discussed at length across all groups. How data was used was important to participants and they were aware of the balance between the need to connect users with professional support and user desires for privacy. However, overall there was a strong push for ***anonymity*** in this context, with specific reference to users feeling “more comfortable” with an app that allowed anonymous use (P1, G1, Female, <18; P4, G1, Female, <18). In relation to ***data usage***, participants were concerned about what would happen to data that were collected by the app, and expressed the importance of explaining this clearly to the user. For example, one participant expressed concern that perhaps data would be “tracked or transferred from one place to another” (P6, G3, Female, 18+), arguing that users would feel more secure using the app if they understood clearly that all information in the app was confidential. However, counter to this, one participant also indicated that over-informing users about the terms of anonymity could result in them feeling “a bit uncomfortable” (P2, G1, Male, <18). This aspect of development requires careful balancing between the clear communication of anonymity, while not becoming overwhelming for the user and thereby risking their disengagement with the app.

Privacy was especially of concern to participants in relation to how they might be connected to professional support services. Participants evinced a preference for being able to log in without using their real names or email addresses, such as through a token system for the first few uses. One participant stated, “Me and my friends can be a little bit dodgy when it comes to signing up for things via email” (P1, G2, Female, 18+).

Similar apprehensions were expressed when it came to the inclusion of features that might enable users to share information (such as about music listening choices) with friends, or with other users. One participant said, “There could be benefits to being able to follow people and stuff, but in general it would kind of complicate the anonymity of it” (P3, G2, Female, 18+). Another from the same group agreed, saying: “If you were going to do that I would want a private option, because I know that in the past, I’ve been judged for music that I’ve listened to” (P1, G2, Female, 18+).

### User empowerment

This theme was identified in relation to several features of the app, including that of prompting help-seeking behaviour. When presented with the idea that the app could track moods over time and then prompt users to seek professional support when it detected an ongoing low mood, participants favoured the idea of allowing users to ***retain choice*** over help-seeking and empowering users to know when to take those steps for themselves.

One participant pointed out that notifications to prompt help-seeking behaviour could actually worsen her mood by reminding her that she was not doing well. She stated, “If someone asked me if I was doing okay it made it all harder, and so if that is a position that somebody is in, they may not appreciate having a reminder of that or getting the data tracked” (P5, G3, Female, 18+). However, another participant did present a contrasting point of view, stating:

Some people don't want to admit to having those kinds of feelings. So I think when they are in a bad mood and it pops up I think that could make them feel better than having to initiate pressing the button to start a chat (P2, G1, Male, <18).

Participants suggested that an alternative to notifications might be to have a prominent icon on all screens of the app enabling users to make their own decisions about when to access support. As one participant put it: “You click on that and you take the action to do it. It’s a bit less like, ‘I’m watching you. Are you okay? Do you need help?’ It’s like you have to take that step which would give you a bit more control” (P4, G4, Female, <18). Another stated, “You don’t really want something coming up and being like, ‘we think you need help’” (P2, G2, Female, 18+). Participants also shared design ideas for such an icon, emphasising that it should express the idea of a “safe space where you can chat”, which would be “a lot more inviting than a crisis text line” (P2, G3, Female, 18+). Design ideas included images such as two hands in the shape of a heart, or something similar to the “care” emoji used on Facebook which depicts a smile emoji hugging a heart. In particular, participants recommended avoiding anything that suggested crisis, a sense of emergency or being forced into contact with health services.

Language use was also very relevant to the idea of user empowerment and control, with participants stressing the importance of any notifications that were received using ***gentle and sensitive language***. Some gave specific suggestions about appropriate wording, such as “Not phrasing that as ‘you need help’, but you should have a more passive take on it like, ‘do you want to chat to a friend?’” (P4, G3, Male, 18+). Another suggested, “Instead of saying things like, ‘you might need help’, or ‘would you like some help?’, maybe the notification could be a bit more light or vague like, ‘would you like to be connected to talk to someone today?’” (P2, G3, Female, 18+).

Customisability was another recurring sub-theme relating to user empowerment. Users valued the ability to set preferences when signing up to the app, so the experience is as tailored as possible to them. One example of this was regarding a suggested visual tool (i.e., a longitudinal plot) through which users could track their moods across time. One participant described wanting the option of being able to opt in or out or whether the app would “track my journey and see how I’m going” (P3, G3, Male, 18+). Another noted that it would be beneficial to have multiple plot options or approaches, “Because I think some people are very different in the way they express how they are feeling” (P3, G2, Female, 18+).

Many participants also preferred that app notifications be customisable. Being able to control the frequency of reminders was important, with several participants noting that too frequent notifications accompanied by sounds could be annoying, especially if they were trying to “zone out and focus on their music” (P5, G4, Male, <18). Participants also requested the functionality to be able to snooze the app, such as if they are listening to music with friends. However, some of these same users were also open to the idea of the app sending a “little check-up” notification in specific situations, such as if they had “a period of really low moods, or they’ve just suddenly gone silent” (P5, G4, Male, <18). This again highlights the balance that mental health apps need to strike between offering assistance, and empowering users to control how and when to engage with the app.

### Engagement vs. achievement

Discussions around motivations and incentives to drive engagement revealed mixed opinions, with some responding well to the idea of incentives such as streaks or achievement rewards, while the majority saw them as too goal-oriented for a mental health app. Some participants favoured small notifications of encouragement, such as, “If they even find in that month three happy songs you can say, ‘congratulations’ … that’s a little bit of hope you could put in there” (P1, G2, Female, 18+). Others liked the idea of stars or badges being awarded to users, purely for aesthetic value, or because it would make them feel excited or happy.

Opposition to the idea of incentives ranged from just saying they were unnecessary, “I don’t really think you need rewards personally” (P4, G1, Female, <18), to outright opposition, “Honestly, I’m quite opposed to it” (P5, G3, Female, 18+). Some felt that the increased skill in mood management would be a reward in itself, “Knowing that you can do better is probably more of a reward than an app saying you’re doing better” (P3, G1, Male, <18). Others felt that it would detract from the purpose of the app and direct attention in the wrong direction. As one participant stated, “I don’t think that having that level thing will help create self-awareness” (P4, G1, Female, <18). Another argued:

I think it's going to turn into this target-based thing where people are going to be benchmarking their performance. I would remove the idea of achievement in that sense because it says that you're trying to work towards something instead of just having the freedom of being and engaging (P5, G3, Female, 18+).

For some, an achievement-based app was something that could contribute to negative mental health outcomes:

Streaks used to be a big thing and that would get everyone on Snapchat in the mornings, and it kind of got a bit unhealthy for a lot of us. We would always feel like if we didn't get that streak we would freak out (P7, G3, Female, 18+).

Another said, “I would respond terribly to badges that had been related to achievement. It’s like I can’t even get out of my bed and you want me to achieve badges” (P5, G3, Female, 18+).

There was general agreement, however, that if any kind of reward system or incentives were implemented, these should celebrate increased skills or efforts to enhance skills rather than mood outcomes, such as “‘you’ve grown the pool of resources that make you happy’, or “just celebrating the fact that you’ve created something to help yourself into a better mood” (P5, G3, Female, 18+).

## Discussion

This study aimed to explore young people's perspectives on encouraging help-seeking behaviour and engagement in a mental health app. Despite recognising the need to promote help-seeking behaviour, participants argued strongly for keeping app usage anonymous, having clear data usage policies, empowering users to make their own choices, using gentle language, and avoiding making mental health apps overly achievement-oriented. Of particular interest in these findings was the suggestion that when aspects of the design were done incorrectly—such as when wording was explicitly directive rather than facilitating autonomous use—a mental health app could in fact have the effect of worsening user wellbeing.

Privacy and anonymity are commonly expressed as concerns by young people in relation to mental health apps in the literature (18, 32). Indeed the American Psychiatric Association includes the need to have a clear and accessible privacy policy and transparency about data usage as a key dimension in its app evaluation model (see for e.g., 33). Nevertheless, a concerning number of mental health apps have unclear policies about data usage or actively use information for other purposes such as targeted advertising (34, 35). A recent review of apps for young people indicated that 39% contained no user-accessible explanation of their privacy policy, and only 50% of the reviewed apps allowed users to delete their data (16). Similarly, a review of mental health apps indicated that only 30% provided a privacy policy (36). As fears surrounding data usage could deter users from accessing the help they need, privacy and anonymity are key concerns that need to be addressed both in app development and in accessible privacy policies.

In general, young people demonstrated mixed viewpoints about the incorporation of incentives into health apps. Prior work has found that reward systems in apps can increase engagement with activities provoking behavioural change (37). Chan, Kow, and Chen (38) found that reward systems were important facilitators of app adoption in young people, yet linking rewards to aspects such as calorie counting or weight tracking was viewed as less desirable. Rather, participants in that study reported feeling that intrinsic motivation that comes from achieving health goals was more important than in-built rewards. This appears to be the same in the context of mental health apps, since participants in the current study viewed it as undesirable to have achievement-based rewards linked to mood outcomes, and some viewed any sort of incentives as giving the wrong message.

In general, intrinsic motivation—engaging in an activity for its own sake rather than for the sake of external reward or to avoid the consequences of not doing so—is widely recognised as being a more preferable model of achievement. Self-determination theory (39) proposes that intrinsic motivation is more likely to lead to sustained behavioural change over time (40). Yet it may be that the perceived desirability of features such as achievement rewards differs depending on an individual's motivational type. Alqahtani and colleagues (2022) found that people with high levels of intrinsic motivation prefer features such as self-monitoring and view rewards as less persuasive. On the other hand, those who tend to be externally motivated or who lack motivation to improve their mental health tend to find rewards more persuasive. Interestingly, Alqahtani and colleagues acknowledged, as did the participants in the current study, that some rewards can have a negative effect on the mood of some users if they feel that they are not achieving enough. Therefore, it may be that optional reward-systems or personalisable motivations may be desirable in mental health apps in order to capture engagement in all users regardless of their motivational type.

Additionally, in line with the findings of existing studies (21, 41), our participants voiced a strong desire to be able to customise various aspects of the app, and to be able to control app settings, including pop-up notifications. While the inclusion of these aspects may well facilitate additional long-term user engagement, many apps require frequent notifications or interactions to achieve their purpose. Successfully navigating these concerns and finding a balance between prompting user engagement while not becoming a burden or irritation to the user provides a significant challenge in the design stages of mental health apps. Our participants also placed clear importance on the ability to postpone or “snooze” notifications altogether when required, such as when they were in a social setting. Considering the embarrassment many young people have about mental health issues, the inclusion of this feature in other mental health apps should not be overlooked.

This study was limited by the fact that health restrictions at the time of data collection due to the COVID-19 pandemic precluded conducting these workshops in person. While online creative collaborations have become more commonplace since the commencement of the pandemic, co-design workshops can benefit from the enhanced communication that comes from face-to-face interactions (42). The study was also limited by the relatively small sample size, and the imbalance of genders across groups. Ideally future studies should further explore the views of males in the 19–25 age group in particular. It should also be noted that since about half of the participants in the groups were not experiencing any form of depression at the time of the study and no information about past experiences was collected, the co-design groups were not wholly constituted by people with lived experience of mental illness. Nevertheless, the findings from this study have helped to illuminate the views of both young people with lived experience of depression and those without. These findings will inform the development of a second iteration of *MoodyTunes,* with further consultation with our co-design groups as development progresses. Further studies will be required to assess the usability, appeal and effectiveness of future iterations of *MoodyTunes* in increasing mental health literacy and the use of helpful mood regulation strategies. Nevertheless, this study highlighted the advantages of a co-design approach, through which we have been able to gain key insights into user preferences for facilitating help-seeking behaviour and improving long-term engagement.

## Data Availability

The datasets presented in this article are not readily available because ethical approval has only been obtained to provide datasets for studies with similar purposes for that which participants have given informed consent. Requests to access the datasets should be directed to the corresponding author.
