# Supplementary material for: Encouraging help-seeking and engagement in a mental health app: What young people want
Source: Front Digit Health. 2022 Dec 21;4:1045765. doi: 10.3389/fdgth.2022.1045765 (PMC9810815; doi:10.3389/fdgth.2022.1045765)
Supplement: Supplementary file 1 [file Datasheet1.docx]

**Focus group discussion points/activities**

1. (Following a visual tour of the prototype of the app)

We will now ask you what you think of the current version of the app.
There are no right or wrong answers. We are interested in what YOU think!

What do you like or dislike?

What would you like to see added or removed?

Does the app look easy to use?

Any other thoughts?

2. We will now show you ideas of what the app might look like in the future.
What do you think? There are no right or wrong answers. We are interested in what YOU think!

What do you like or dislike?

What would you like to see added or removed?

Any other thoughts?

3. (Visual examples from other apps were shown to facilitate this discussion)

How can we best allow users to access emergency help in this app?

What kind of visualisations of mood changes over time would you like to see in the app? What should such a visualisation depict?

What kind of incentives/levels/gamification would be fun for users?
